# Direct administration of human leucocyte antigen (dHLA) molecules into tumour sites: proposal for a new immunotherapy for cancer

**DOI:** 10.1038/s44276-023-00017-7

**Published:** 2023-10-23

**Authors:** Apostolos P. Georgopoulos, Lisa M. James

**Affiliations:** 1 The HLA Research Group, Brain Sciences Center, Department of Veterans Affairs Health Care System, Minneapolis, MN 55417 USA; 2 Department of Neuroscience, University of Minnesota Medical School, Minneapolis, MN 55455 USA

## Abstract

Natural elimination of cancer cells is mediated by human leucocyte antigen (HLA) Class I and II molecules [1] that bind to tumour protein fragments, move to the tumour cell surface and engage CD8+ lymphocytes for killing the tumour cell (HLA Class I) and CD4+ lymphocytes for antibody production against the tumour cell (HLA Class II). HLA Class I molecules are present in all nucleated cells, whereas Class II molecules are present only in antigen presenting cells, such as macrophages. These defence mechanisms are suppressed by tumour cells; this tumour-induced immunosuppression is partly reversed by immune checkpoint inhibitors (ICI), thus allowing the HLA-based tumour elimination to be active again; indeed, the success of ICI therapy partly depends on the HLA makeup of the recipient [2, 3]. Here, we propose a novel cancer immunotherapy consisting of administering the mRNA blueprints for the synthesis of specific HLA Class I molecules with high binding affinity and high immunogenicity to a tumour’s neoantigens. This should maximise the effectiveness of HLA-mediated tumour elimination.

## Background

### HLA and cancer

It is increasingly recognised that human leucocyte antigen (HLA) significantly influences cancer susceptibility, prognosis, and immunotherapy effectiveness [2–4]. HLA Class I and II are cell-surface molecules that bind with and present tumour antigens to CD8+ and CD4+ T cells to signal cell death and antibody production, respectively. The success of this natural antitumor response lies, in part, in the binding affinity and immunogenicity of the HLA-antigen complex. The HLA region is the most highly polymorphic of the human genome; consequently, HLA composition varies tremendously across individuals. Yet each individual possesses only 12 classical HLA genes, influencing and limiting the repertoire of antigens that can be bound and transported to the cell surface for T-cell presentation. In the absence of an HLA-antigen match and/or insufficient immunogenicity, the ability to mount an immune response to facilitate natural tumour elimination is hindered.

### Immunosuppression by tumour cells

An additional consideration with regard to the effectiveness of tumour elimination is that the immune response above may be hindered due to immunosuppression by tumour cells [5]. Cancer-induced alteration or loss of HLA expression inhibits immune detection and subsequent activation of the immune system thereby permitting immune escape and proliferation of cancer cells. HLA alterations also adversely impact clinical response to immunotherapy; consequently, therapeutic strategies aimed at enhancing the immune response to induce antitumor immunity are of great interest. Two strategies that have shown promise are immune checkpoint inhibitors and neoantigen-based cancer vaccines.

### Cancer neoantigens

During carcinogenesis, nonsynonymous somatic mutations result in the formation of neoantigens. Tumour-specific neoantigens are expressed only on tumour cells and are considered non-self by the immune system, prompting activation of the immune response to attack cancer cells [6]. Consequently, there is increasing interest in targeting tumour-specific neoantigens via personalised vaccine immunotherapy.

### Immunotherapy using immune checkpoint inhibitors (ICI)

One promising cancer immunotherapeutic strategy is the use of immune checkpoint inhibitors. Tumour cells express immune-suppressing proteins known as immune checkpoints that inhibit T-cell activation. Immune checkpoint inhibitors block immune suppression and trigger the host antitumor immune response [5]. The clinical effectiveness of immune checkpoint inhibitors, however, is dependent on HLA phenotype [2, 3, 5].

### Immunotherapy using neoantigens

An alternative cancer immunotherapeutic strategy is personalised neoantigen-based immunotherapy which involves the administration of cancer vaccines (e.g., messenger RNA (mRNA) vaccines, DNA vaccines, peptide vaccines, dendritic cell vaccines) to stimulate immune activation against tumour-specific neoantigens [6, 7]. Though clinical trials are encouraging, there remain several challenges and limitations of this approach [6]. One key limitation is the assumption that the patient is able to mount an antitumor immune response as a result of vaccine administration. As discussed above, successful engagement of the immune system rests on the formation of a specific HLA molecule-antigen complex which requires the presence of HLA molecules that can bind with sufficient affinity and immunogenicity to the neoantigen. The ability to form an HLA-antigen complex is limited by the HLA genes that an individual carries.

### Summary

Whether natural or via immunotherapy, an effective antitumor response requires the presence of HLA molecules capable of binding tumour antigens with high immunogenicity. The capability of forming an HLA-tumour antigen complex that can stimulate an antitumor immune response is limited by the small number of HLA molecules that each individual possesses.

## A new immunotherapy for cancer: dHLA

Here, we propose that providing to the patient with the HLA molecule(s) suitable for effective binding to tumour neoantigens would aid in tumour cell elimination in conjunction with ICI therapy.

### Rationale

Our proposal above addresses only HLA Class I molecules and rests on the following assumptions and rationale.

- Neoantigens that bind to HLA Class I molecules aid tumour cell elimination by engaging CD8+ (HLA Class I) T cells to kill tumour cells containing the neoantigen(s).
- Given the immense polymorphism of HLA genes, it is very likely that there exist HLA Class I molecules with high binding affinity to neoantigens and sufficient T-cell immunogenicity of the HLA molecule-neoantigen complex to aid tumour cell elimination with the aforementioned mechanism. Such HLA molecules can be identified using information from various sources [6].
- Since a particular individual carries only 6 HLA classical Class I genes (3 doublets from A, B, C genes), there is only a limited chance that specific neoantigens would form complexes with HLA molecules of sufficient immunogenicity to be substantially effective in tumour cell elimination. This is reflected in the partial success of ICI and/or neoantigen mRNA administration therapies, this being partly due to the fact that even in the absence of tumour-induced immunosuppression (in ICI) and/or the presence of specific neoantigens (via mRNA administration), the outcome critically depends on the availability of suitable HLA molecules to form an effective complex with the neoantigen.
- Here we propose a solution to this problem, namely to provide the individual with the suitable HLA molecules to form effective complexes with neoantigens leading to successful CD8+ T lymphocyte engagement for tumour cell elimination via direct killing (CD8+) of tumour cells.

### Providing HLA molecules suitable for neoantigen binding

We envision the following steps in implementing our procedure.

- Identification of neoantigens for specific cancers. There is substantial information on this already in the literature [6, 8, 9] and can be obtained from the tumour of a specific patient.
- Identification of HLA Class I molecules that bind with high affinity to, and possess high immunogenicity against, the cancer neoantigens above. Such alleles can be identified directly using in silico methods [8] and/or from the outcomes of ICI therapy that depend on the patients’ HLA makeup [2–4], or from other sources [9].
- Construction of mRNA blueprint(s) of the selected HLA Class I molecules. This is feasible given that (i) the amino acid sequences of HLA Class I molecules are known, and (ii) complete tetramer HLA molecules are currently being synthesised in vitro [10, 11] and shown to bind various peptides [11].
- The technology of inducing the synthesis of proteins by administering their mRNA blueprint is well advanced [12].

### Implementation

We expect the implementation of our proposed dHLA therapy to be straightforward.

**Fig. 1:**
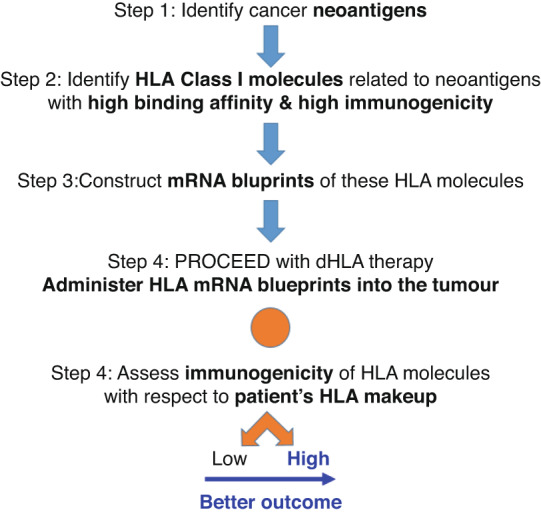
Proposed direct HLA (dHLA) immunotherapy for cancer. Steps in choosing dHLA, assessing dHLA potential risk, and implementing implement it. HLA, Human Leucocyte Antigen; mRNA, messenger ribonucleic acid.

- The therapeutic HLA molecules will be administered to tumour sites. HLA Class I molecules can be synthesised by all nucleated cells, and, hence, by cancer cells. Therefore, a successful reduction and/or elimination of the tumour cells is to be expected, assuming that tumour-induced immunosuppression is absent or low (e.g., at very early stages of tumour development) or has been reduced/lifted by using, for example, ICI therapy.
- It is well known that HLA molecules can be immunogenic, i.e., antibodies can be produced against them leading to a variety of adverse effects, including the rejection of a transplanted organ. Remarkably, this may work to the advantage of our proposed dHLA immunotherapy because such an effect will enhance the elimination of the tumour, which, in this case, will behave as a foreign body to be rejected. In fact, in a reverse consideration, therapeutic HLA molecules with high immunogenicity would be more potent in tumour elimination: whereas in the transplant case, organ donor(s) are sought with a similar HLA makeup as the organ recipient, in our case, one would strive to select cancer neoantigen-specific HLA molecules with high incompatibility to the HLA of the patient.
- The local administration of the therapeutic HLA molecules ensures the localisation of the effect on the tumour site(s) and the absence of generalised side effects.
- Given the enormous polymorphism of HLA, it is reasonable to expect that several cancer-specific HLA molecules will be identified from which to choose those with high binding affinity and T-cell engagement [13] and, potentially, of high incompatibility with the HLA of the patient, resulting in successful tumour reduction/elimination. In fact, a number of mRNA HLA blueprints can be injected into the tumour simultaneously to maximise the tumour elimination effect.
- The treatment could be repeated many times.
- The principles of dHLA therapy and the steps of its implementation are presented in Fig. 1. We believe that dHLA cancer therapy has the potential to become an effective new tool in the fight against cancer.

### Limitations

- The main limitation of the proposed therapy is its application to solid tumours and/or tumour sites accessible for direct administration of the mRNA HLA blueprints.
- This treatment could be used for metastases or tumour sites that are difficult to access if administered systemically. However, in this case, generalised side effects may appear that will need to be evaluated and treated according to the medical condition of the patient.
